# The "Goutte De Lait"

**Published:** 1904-10-01

**Authors:** 


### HOSPITAL ADMINISTRATION.

### CONSTRUCTION AND ECONOMICS.

### THE "GOUTTE DE LAIT."

(*Continued from Vol. XXXVI., page* 452.)

In the Belleville "Goutte de Lait," which may be considered typical, every case is treated individually. On the occasion of the first visit the child is weighed, and careful note is made of its condition. Treatment is prescribed, medicinal if desirable, and if the mother is unable to suckle it she receives a free gift of sterilised milk for its nurture. It is brought back in a week, the weight is again taken, another examination is made and a comparison with the former record shows if progress has been made, and if so to what extent. The treatment is modified according to the result. And every week the séance is repeated. Every week the highest French medical skill in the treatment of infant maladies is placed at the service of those too poor to pay the humblest doctor's fee. This continues until the nursling has passed the age of suckling, which, it may be remarked, is in France usually continued past the time when a British baby is held to have reached the stage for more solid food than milk.

No........................

Name ..............................

Address ..........................

Date of Birth..................... Sex...............

| Date | Weight | Gain or Loss | Quantity of Milk | Observations |
|---|---|---|---|---|
| | | | | |

The card upon which particulars of the child's condition and treatment are registered week by week is spaced as shown in the accompanying illustration. An examination of some of these cards in the Belleville dispensary throws a strong light upon the deplorable condition of many of the children brought for treatment. One which now lies before the writer shows that a female child of over eight months old was carried to Dr. Variot in April 1903 with a broken thigh, suffering from gastro-enteritis and weighing only 10 lb. Increase in weight was at first slow on account of the debility with which the little frame was struggling. After three months it had gained only 1 lb., but after a year it turned the scales at 21 lb. and was in sound health. Of course dietary alone did not save the child's life. Medical treatment played its part but the dietary was not less important and carried the little sufferer so far towards normal health. The instance is cited not as evidence in favour of sterilised milk but to show the field in which the "Gouttes de Lait" operate. The subjects of the charities' benevolence are the poorest of the Parisian poor, and patients in conditions as low as that mentioned are far too numerous.

The feeding-bottle given for the use of the child is of a special type introduced by Dr. Variot. It is graduated in a special manner indicating by raised letters and figures how many times a day the child ought to be fed, whatever its age, and how much it ought to receive each time. In special cases the average requirements represented by the figures are departed from under the advice of the doctors, as naturally the nurture of children cannot be governed mathematically and every child of equal age is not suited for the same amount of food.

The amount of work accomplished by the Belleville "Goutte de Lait" is, to some extent, computable in figures. During 10 years more than 400,000 litres of sterilised milk have been distributed to more than 3,000 nurslings. As the children are examined each week these figures represent something like 100,000 examinations, no mean record of honourable and honorary work.

The Belleville "Goutte de Lait" has had many imitators. In Paris alone there are now 25 institutions operating on similar lines. By their united efforts watch is kept over 2,000 infants born each year until the months of early infancy have passed. There remain 38,000 infants which do not come into touch with the institutions. It is a conservative estimate that two-thirds of the entire infant population would derive material benefit if they were subject to the surveillance exercised over the five per cent. whom the "Gouttes de Lait" influence. The agencies must thus be multiplied or strengthened more than ten-fold before they have reached the ability proportionate to the occasion. Private charity, the reed upon which they now lean, cannot hope to provide for this increase.

Throughout provincial France there are several towns where the "Goutte de Lait" has taken root, and, including those in Paris, the country contains 65 such charities. Two of the most important are in Rouen and in Saint-Pol-sur-Mer near Dunkirk. In both towns the Gouttes de Lait found conditions upon which they wrought a wonderful improvement. In Rouen the death-rate of infants under one year old was 33 per cent. of births, and in Saint-Pol-sur-Mer it averaged 28·8 per cent. for the five years ending 1901. The year 1902-1903 saw a decrease in the latter town to 20·9 per cent. and the "Goutte de Lait" is credited with having been the most active factor in achieving this result. Throughout France the average death-rate during the first year of life is 16½ per cent. of the births.

Statistics show that in our own England the mortality of infants before they have reached the age of one year is little short of that in France, and much higher than in many other European countries. Dr. Johannessen, of Stockholm, vouches for the accuracy of the following figures:—

| | | |
|---|---|---|
| Norway ... ... ... ... | 9 5 | per cent. |
| Sweden ... ... ... ... | 9 8 | " |
| Ireland ... ... ... ... | 10 6 | " |
| Denmark... ... ... ... | 12 8 | " |
| England ... ... ... ... | 15 0 | " |
| France ... ... ... ... | 16·5 | " |
| Italy ... ... ... ... | 16·7 | " |

While the reasons which compel attention to our own country are not similarly urgent to those operating in France, yet humanitarian and social considerations call for attempts to reduce the death-rate among young children. To have to admit that in England three in every 20 children die before the first anniversary of birth is humiliating, and the confession brings into the light one department which our vaunted progress in medical and hygienic science seems to have influenced little. The congested quarters in our cities are the pest holes where the tribute of infant life is the highest. Poverty, ignorance, carelessness, and crime are the high priests that officiate before the sacrificial altars.

The "Goutte de Lait" movement of France has sent some seeds across the Channel, and there are in Great Britain six institutions—in Battersea, Liverpool, St. Helens, Bradford, York, and Leith—more or less resembling the French charities. But it will be so long before this little leaven can permeate the whole lump that one is constrained to lift a loud voice, and after proclaiming the urgency of the need, to try to show the particular manner in which the most desirable results may be won. In so many of the details—the cows, the milk, its preparation, its distribution, and its administration—is it possible to adopt methods which fall short of the highest. Healthy cows, properly fed and cleanly lodged, are the first essentials, and stringency in concern for these conditions can scarcely err on the side of too great severity. The method of milking—the new mechanical method by suction from an electrically-driven apparatus should be tested in all its aspects—and the storing of the milk follow. Contamination is so easy that every stage must be watched. Scrupulous care in one direction may easily be negatived by laxity in another. Then the preparation: Sterilisation or Pasteurisation—popularly confounded, but greatly different—the advisability of pure milk, of diluted milk, or of modified milk are all questions that need the careful consideration of experienced medical men. It is in the distribution that the most stubborn difficulties have to be encountered. Municipal or State aid is absolutely necessary to the success of the schemes. Their prosecution cannot be wide enough otherwise.

In Liverpool and Battersea we have municipal milk distribution, but it is without that direct and skilled medical supervision which is essential to the highest good. It is better far than no scheme at all, but it may be raised to a higher plane by a slight rearrangement of the control. Several suggestions offer in this department. One is that any qualified doctor's order for municipal milk should be honoured at the distributing agency. This would enable every child to remain under the care of its own medical attendant, a most important consideration. The recognition of such orders, as a feature, would at once place the scheme upon a broad and solid foundation of usefulness. To what extent the law ought to be invoked to make periodical examination of the health of infants compulsory, and to insist upon the observance of the prescribed directions, is a proper matter for consideration. The suggestion may appear extreme, but is not the need sufficiently urgent? Regard the average infant mortality already noticed. Three in 20 children die before the age of 12 months. This is the average. Double it, make it 3 in 10, and you are still under the figure for the worst quarters in our cities. And this is without reckoning the deaths over one year old, for which neglect during the first year is directly responsible. Compulsion in the direction indicated would only be carrying the aims of the promoters of legislation against child labour higher and the purposes behind free education deeper. To preserve the infant life to fill its destiny in the State is a national duty, and as important a work as education, the burden of which the State shoulders. *Corpus sanum* ssential and antecedent to *mens sana.*

